# Robust Smoothing Cardinalized Probability Hypothesis Density Filter-Based Underwater Multi-Target Direction-of-Arrival Tracking with Uncertain Measurement Noise

**DOI:** 10.3390/e27040438

**Published:** 2025-04-18

**Authors:** Xinyu Gu, Xianghao Hou, Boxuan Zhang, Yixin Yang, Shuanping Du

**Affiliations:** 1 School of Marine Science and Technology, Northwestern Polytechnical University, Xi’an 710072, China; guxinyu@mail.nwpu.edu.cn (X.G.); yxyang@nwpu.edu.cn (Y.Y.); 2 Hangzhou Applied Acoustic Research Institute, Hangzhou 310023, China; dushuanping@163.com; 3 Shaanxi Key Laboratory of Underwater Information Technology, Xi’an 710072, China; 4 Xi’an Precision Machinery Research Institute, Xi’an 710077, China; bxzhang_uw@outlook.com

**Keywords:** underwater multi-target direction-of-arrival tracking, uncertain measurement noise, spatial matrix filter, cardinalized probability hypothesis density filter

## Abstract

In view of the typical multi-target scenarios of underwater direction-of-arrival (DOA) tracking complicated by uncertain measurement noise in unknown underwater environments, a robust underwater multi-target DOA tracking method is proposed by incorporating Saga–Husa (SH) noise estimation and a backward smoothing technique within the framework of the cardinalized probability hypothesis density (CPHD) filter. First, the kinematic model of underwater targets and the measurement model based on the received signals of a hydrophone array are established, from which the CPHD-based multi-target DOA tracking algorithm is derived. To mitigate the adverse impact of uncertain measurement noise, the Saga–Husa approach is deployed for dynamic noise estimation, thereby reducing noise-induced performance degradation. Subsequently, a backward smoothing technique is applied to the forward filtering results to further enhance tracking robustness and precision. Finally, extensive simulations and experimental evaluations demonstrate that the proposed method outperforms existing DOA estimation and tracking techniques in terms of robustness and accuracy under uncertain measurement noise conditions.

## 1. Introduction

DOA tracking aims to estimate the bearing angles of moving targets in real time. Conventional methods [1,2,3,4] either employ raw signals received by an array or bearing estimates from traditional DOA [5,6,7] techniques as measurements. With the aid of a kinematic model for the target’s bearing and an appropriate measurement model, Bayesian filtering is used to recursively estimate the target bearing. By combining measurement data with the dynamic characteristics of underwater targets, DOA tracking methods substantially improve estimation accuracy. In complex and uncertain underwater environments, additional measurement noise and interference make state inference more challenging and may reduce estimation precision. To tackle these challenges, Bayesian filtering continuously incorporates new measurements to iteratively refine the posterior distribution of the target state, thereby enhancing estimation reliability and tracking robustness, even under adverse conditions.

Early research on tracking algorithms mainly focused on single-target scenarios. Initially, bearing estimates obtained from traditional DOA estimation methods were used as measurements, which led to the development of DOA tracking algorithms based on the Kalman Filter (KF). The linear relationship between these measurements and bearing angles facilitated effective single-target tracking. However, when array signals were employed directly as measurements, the relationship became nonlinear, resulting in nonlinear measurement models. To address this challenge, researchers introduced various nonlinear Bayesian filtering techniques, such as the Extended Kalman Filter (EKF) for linearizing nonlinear models, the Unscented Kalman Filter (UKF), the Cubature Kalman Filter (CKF), and Particle Filtering (also known as Sequential Monte Carlo or SMC methods), which represents the target distribution using a large number of particles to better handle nonlinearity. For instance, Kong [8] successfully tracked target bearings using the EKF with array signal measurements, while Orton [9] enhanced tracking accuracy through particle filtering by deriving the likelihood function for array signal measurements, albeit at the cost of higher computational demand.

Methods for addressing multi-target tracking problems can be broadly classified into two categories as follows: traditional data association methods and random finite set (RFS)-based approaches. Data association methods work by establishing correspondences between measurements and targets, effectively reducing the multi-target tracking challenge to a set of single-target tracking problems. Although effective, these methods are computationally intensive. Since the early 21st century, RFS-based approaches—which model the collection of targets and measurements as random finite sets with both random elements and random cardinality—have experienced rapid development. These approaches avoid the need for explicit measurement-to-target associations, thereby significantly reducing computational complexity. In the context of multi-target tracking, Mahler [10] introduced the concept of “first-order moment filtering”, commonly known as Probability Hypothesis Density (PHD) filtering.

In multi-target tracking, uncertainty arises in both target existence and state estimation. The PHD filter, by propagating the first-order moment of the target state probability density, effectively reduces this uncertainty and improves tracking accuracy. Similarly, CPHD filtering extends the idea by incorporating cardinality estimation to further minimize uncertainty regarding the number of targets. Clark et al. [11] employed a Gaussian Mixture (GM) model to represent the PHD under the assumption of linear Gaussian target motion and measurement models, leading to the development of Gaussian Mixture Probability Hypothesis Density (GM-PHD) filtering that successfully bridges theory and practice for multi-target tracking. Vo et al. [12] introduced Sequential Monte Carlo PHD filtering (SMC-PHD), which utilizes particle filtering to track multiple targets under nonlinear models; however, particle-based implementation requires significant computational resources. Later, Mahler [13] advanced PHD filtering by incorporating cardinality distribution to model the target count, resulting in the development of Cardinalized Probability Hypothesis Density (CPHD) filtering that more accurately estimates the number of targets and further enhances tracking performance. As with PHD filtering, CPHD filtering does not have a universal closed-form solution and must be implemented via SMC methods or a GM model. Given that random finite set-based approaches generally offer lower computational complexity compared to traditional data association methods, researchers have proposed multi-target bearing tracking techniques based on this framework. These techniques use array signal measurements to achieve accurate multi-target bearing tracking under nonlinear measurement models with the SMC approach. Nevertheless, due to the high dimensionality of array signal measurements and the extensive number of particle states required by the SMC method, such multi-target bearing tracking techniques remain computationally intensive.

In real underwater target bearing tracking scenarios, in addition to the target’s own acoustic signals, the ocean environment introduces additional noise due to factors such as ocean turbulence, distant ships, distant storms, and surface waves, all of which can adversely affect the measurement of target bearings. In the Bayesian filtering framework, this manifests as uncertainty in the measurement noise parameters; if the variance parameters are not accurately specified, tracking performance can be significantly degraded. To cope with this time-varying noise, researchers have developed adaptive filtering techniques that estimate noise statistics in real time and incorporate these estimates into the Bayesian filtering process to enhance tracking performance. Noise-adaptive estimation techniques, such as the Saga–Husa algorithm, dynamically adjust the noise parameters to reduce the detrimental effects of time-varying measurement noise. For single-target bearing tracking, Zhang et al. [3] employed an improved Saga–Husa algorithm to estimate the covariance matrix of the measurement noise and proposed the SH-AEKF method, achieving robust underwater single-target DOA tracking under uncertain measurement noise conditions. In addition, by utilizing the Variational Bayesian (VB) method to jointly estimate target bearings and the noise covariance matrix, the VB-AEKF method was developed, further enhancing tracking accuracy [14,15,16]. Building upon these results, researchers [17,18] have extended these noise-adaptive techniques to the multi-target scenario. Within the Gaussian Mixture Cardinalized Probability Hypothesis Density (GM-CPHD) filtering framework, the Saga–Husa algorithm and the VB method have been adapted for multi-target applications, leading to the development of the SH-CPHD and VB-CPHD filtering methods for robust underwater multi-target DOA tracking.

Methods based on SH and VB estimation improve underwater target bearing tracking under uncertain noise. Yet, errors occur when measurement noise variance changes suddenly. To boost robustness in multi-target bearing tracking, we use SH-CPHD for forward filtering. Then, we apply backward smoothing to process the results. This yields the smoothed SH-CPHD filtering method. This method is built on the GM-CPHD framework. It uses target bearings from traditional DOA methods as measurements. Measurement bias is modeled as zero-mean Gaussian noise. This models the uncertain noise from unknown ocean acoustics. The uncertain noise worsens tracking accuracy. To fix this, we use the Saga–Husa algorithm for real-time noise variance estimation. This reduces the noise effect and improves tracking robustness. Backward smoothing further reduces errors by refining the state estimates. Simulation and field data show that, compared to other methods, our approach estimates noise variance well. This minimizes uncertainty and greatly improves tracking accuracy.

The structure of this paper is as follows: Section 2 introduces the motion and measurement models for multi-target scenarios; Section 3 presents the CPHD based multi-target DOA tracking algorithm; Section 4 further introduces the SH-CPHD algorithm; Section 5 provides a detailed description of the proposed method based on Section 3 and Section 4; Section 6 validates and analyzes the proposed algorithm through simulation experiments; Section 7 verifies the practicality and robustness of the proposed algorithm in real ocean environments using the publicly available SWellEx96 dataset, demonstrating its superiority over several alternative methods; and finally, Section 8 concludes the paper.

## 2. Multi-Target Tracking Model

### 2.1. Kinematic Model of the Underwater Target by the Bearing Angle

The sets of the target states are assumed to be RFSs. Assuming that $n_{k}$ targets exist within the detection area of the sonar at time step *k*, and the state of the $i_{n}$-th target is $x_{k}^{i_{n}}$, ($i_{n}=1,2,\cdots ,n_{k}$), the set of the targets’ states at time step *k* is expressed as $X_{k}=\{x_{k}^{1},x_{k}^{2},\ldots ,x_{k}^{n_{k}}\}$. Let $\theta_{k}$ denote the bearing angle of the $i_{n}$-th target ($\theta_{k}^{i_{n}}$ is the angle between the target and the positive *x*-axis with respect to the positive counterclockwise.) and let ${\dot{\theta }}_{k}^{i_{n}}$ denote the change rate of $\theta_{k}^{i_{n}}$. The state of the $i_{n}$-th target is expressed as $x_{k}^{i_{n}}={\left(\theta_{k}^{i_{n}},{\dot{\theta }}_{k}^{i_{n}}\right)}^{T}$, where ${\left(\cdot \right)}^{T}$ denotes the matrix transposition. Since the underwater targets typically do not maneuver to save energy and remain concealed, the bearing angles are assumed to follow a constant velocity (CV) model. The CV model of the state of the $i_{n}$-th target is expressed as follows:

(1) $$\left\{ \begin{array}{l} x_{k}^{i_{n}}=F_{k|k-1}x_{k-1}^{i_{n}}+G_{k}w_{k}\\ F_{k|k-1}=\left[\begin{array}{cc} 1 & T\\ 0 & 1 \end{array}\right],\;G_{k}=\left[\begin{array}{c} T^{2}/2\\ T \end{array}\right] \end{array}\right.$$

Where $F_{k|k-1}$ and $G_{k}$ are the state transition matrix and the noise driving matrix; $w_{k}$ denotes the zero-mean Gaussian process noise with the covariance matrix $Q_{k}$, and *T* is the interval between the adjacent time steps.

### 2.2. Measurement Model

It is posited that $m_{k}$ targets are identified at time step *k*. Due to the presence of detection probability and false alarm rate, the count of measurements and the states of targets always vary in a multi-target tracking scenario. It is postulated that the $m_{k}^{d}$ target is detected along with $m_{k}^{f}$ false alarms $\left\{z_{k}^{1},z_{k}^{2},\ldots ,z_{k}^{m_{k}^{f}}\right\}$, resulting in $m_{k}$ ($m_{k}=m_{k}^{d}+m_{k}^{f}$) measurements at time step *k*. The false alarms $\left\{z_{k}^{1},z_{k}^{2},\ldots ,z_{k}^{m_{k}^{f}}\right\}$ are presumed to follow a Poisson distribution with an intensity of $\kappa_{k}$. The collection of measurements at time step *k* is denoted as $Z_{k}=\{z_{k}^{1},z_{k}^{2},\ldots ,z_{k}^{m_{k}}\}$ and is also considered an RFS. The measurement corresponding to the $i_{m}^{d}$-th detected target $z_{k}^{i_{m}^{d}}$ at time step *k* is represented as follows:

(2) $$z_{k}^{i_{m}^{d}}=H_{k}x_{k}^{i_{m}^{d}}+v_{k}$$

Where $H_{k}$ denotes the measurement matrix and $H_{k}=[1,0]$. $v_{k}$ represents the bearing angle measurement noise, which must be distinguished from other types of measurement noise. $v_{k}$ is the error in the bearing angle estimation obtained using traditional DOA estimation methods. We assume it follows a Gaussian distribution with a mean of zero and a variance of $\sigma_{r,k}^{2}$.

In a real underwater tracking scenario, the unknown conditions beneath the surface may lead to fluctuations in the variance $\sigma_{r,k}^{2}$, a challenge that traditional tracking methods struggle to manage. This difficulty significantly reduces the efficacy of these traditional tracking techniques.

## 3. GM-CPHD Filter for Multi-Target DOA Tracking

Multi-target tracking is performed by the CPHD filter through the recursive calculation of the PHD and the cardinality distribution, representing the distribution of the states and the number of targets, respectively. The closed-form solution of the CPHD filter is provided under the assumption of a linear Gaussian mixture model, referred to as the GM-CPHD filter. Each component of the Gaussian mixture model represents a target’s state. The algorithm for the GM-CPHD recursion is then provided as follows:

At time k≥1

*Step* 1. *Prediction*

Given the cardinality distribution $p_{k-1}\left(n\right)$ at time step *k* − 1, the predicted cardinality distribution is described as follows:

(3) $$p_{k|k-1}\left(n\right)=\sum_{j=0}^{n}p_{\Gamma ,k}\left(n-j\right)\sum_{l=j}^{\infty }C_{l}^{j}p_{k-1}\left(l\right)p_{s,k}^{j}{\left(1-p_{s,k}\right)}^{l-j},$$

Where $C_{l}^{j}=l!/\left(j!\left(l-j\right)!\right)$, $p_{\Gamma ,k}\left(\cdot \right)$ represents the cardinality distribution of birth targets, and $p_{s,k}$ denotes the probability of targets surviving.

Given the PHD $v_{k-1}\left(x\right)$ at time step *k* − 1, the predicted PHD can be represented as follows:

(4) $$v_{k|k-1}\left(x\right)=v_{S,k|k-1}\left(x\right)+\gamma_{k}\left(x\right),$$

Where $v_{S,k|k-1}\left(x\right)$ denotes the PHD of surviving targets, and $\gamma_{k}\left(x\right)$ denotes the PHD of birth targets. $v_{S,k|k-1}\left(x\right)$ can be represented as follows:

(5) $$v_{s,k|k-1}\left(x\right)=p_{s,k}\sum_{j=1}^{J_{k-1}}w_{k-1}^{\left(j\right)}N\left(x;m_{s,k|k-1}^{\left(j\right)},P_{s,k|k-1}^{\left(j\right)}\right)$$

Where $m_{s,k|k-1}^{\left(i\right)}$ and $P_{s,k|k-1}^{\left(i\right)}$ denote the predicted state and the predicted MSEM of surviving targets, respectively. $m_{s,k|k-1}^{\left(i\right)}$ and $P_{s,k|k-1}^{\left(i\right)}$ are given as follows:

(6) $$m_{s,k|k-1}^{\left(j\right)}=F_{k-1}m_{k-1}^{\left(j\right)}$$

(7) $$P_{s,k|k-1}^{\left(j\right)}=G_{k}\sigma_{q}^{2}G_{k}^{T}+F_{k-1}P_{k-1}^{\left(j\right)}F_{k-1}^{T}$$

$\gamma_{k}\left(x\right)$ in Equation (4) is subject to the Gaussian mixture model as follows:

(8) $$\gamma_{k}\left(x\right)=\sum_{i=1}^{J_{\gamma ,k}}w_{\gamma ,k}^{\left(i\right)}N\left(x;m_{\gamma ,k}^{\left(i\right)},P_{\gamma ,k}^{\left(i\right)}\right).$$

Where $w_{\gamma ,k}^{\left(i\right)}$ denotes the weight, and $m_{\gamma ,k}^{\left(i\right)}$ and $P_{\gamma ,k}^{\left(i\right)}$ are the state and the MSEM of the birth target, respectively.

According to Equations (4), (5) and (8), the predicted PHD is expressed in the form of a Gaussian mixture model as follows:

(9) $$v_{k|k-1}\left(x\right)=\sum_{i=1}^{J_{k|k-1}}w_{k|k-1}^{\left(i\right)}N\left(x;m_{k|k-1}^{\left(i\right)},P_{k|k-1}^{\left(i\right)}\right)$$

Where $w_{k|k-1}^{\left(i\right)}$ denotes the weight, $m_{k|k-1}^{\left(i\right)}$ denotes the predicted state, and $P_{k|k-1}^{\left(i\right)}$ denotes the predicted MSEM of the target.

*Step* 2. *Update*

The cardinality distribution $p_{k}\left(n\right)$ and the PHD $v_{k}\left(x\right)$ at time step *k* are derived by updating the predicted cardinality distribution $p_{k|k-1}\left(n\right)$ and the predicted PHD $v_{k|k-1}\left(x\right)$ using the measurement set $Z_{k}$ as follows:

(10) $$p_{k}\left(n\right)=\frac{\Psi_{k}^{0}\left[w_{k|k-1},Z_{k}\right]\left(n\right)p_{k|k-1}\left(n\right)}{\langle \Psi_{k}^{0}\left[w_{k|k-1},Z_{k}\right],p_{k|k-1}\rangle }$$

(11) $$\begin{array}{ll} v_{k}\left(x\right)= & \frac{\langle \Psi_{k}^{1}\left[w_{k|k-1},Z_{k}\right],p_{k|k-1}\rangle }{\langle \Psi_{k}^{0}\left[w_{k|k-1},Z_{k}\right],p_{k|k-1}\rangle }\left(1-p_{D,k}\right)v_{k|k-1}\left(x\right)\\ & +\sum_{z\in Z_{k}}\sum_{j=1}^{J_{k|k-1}}w_{k}^{\left(j\right)}\left(z\right)N\left(x;m_{k}^{\left(j\right)},P_{k}^{\left(j\right)}\right) \end{array}$$

Where 〈α,β〉 denotes the inner product of α and β, i.e., $\langle \alpha ,\beta \rangle =\sum_{l=1}^{L}\alpha_{l}\beta_{l}$ ($\alpha =\left[\alpha_{1},\alpha_{2},\ldots ,\alpha_{L}\right]$, $\beta =\left[\beta_{1},\beta_{2},\ldots ,\beta_{L}\right]$), $p_{D,k}$ denotes the detection probability, and

(12) $$\Psi_{k}^{u}\left[w,Z\right]\left(n\right)=\sum_{j=0}^{\min\left(\left|Z\right|,n\right)}\left(\left|Z\right|-j\right)p_{K,k}\left(\left|Z\right|-j\right)A_{j+u}^{n}\frac{{\left(1-p_{D,k}\right)}^{n-\left(j+u\right)}}{{\langle 1,w\rangle }^{j+u}}e_{j}\left(\Lambda_{k}\left(w,Z\right)\right)$$

(13) $$\Lambda_{k,z}\left(x\right)=\left\{\frac{\langle 1,\kappa_{k}\rangle }{\kappa_{k}\left(z\right)}p_{D,k}w^{T}q_{k}\left(z\right):z\in Z\right\}$$

(14) $$w_{k|k-1}={\left[w_{k|k-1}^{\left(1\right)},\ldots ,w_{k|k-1}^{\left(J_{k|k-1}\right)}\right]}^{T}$$

(15) $$q_{k}\left(z\right)={\left[q_{k}^{\left(1\right)}\left(z\right),\ldots ,q_{k}^{\left(J_{k|k-1}\right)}\left(z\right)\right]}^{T}$$

(16) $$q_{k}^{\left(j\right)}\left(z\right)=N\left(z;\eta_{k|k-1}^{\left(j\right)},S_{k|k-1}^{\left(j\right)}\right)$$

(17) $$\eta_{k|k-1}^{\left(j\right)}=H_{k}m_{k|k-1}^{\left(j\right)}$$

(18) $$S_{k|k-1}^{\left(j\right)}=H_{k}P_{k|k-1}^{\left(j\right)}H_{k}^{T}+\sigma_{r,k}^{2}$$

(19) $$w_{k}^{\left(j\right)}\left(z\right)=p_{D,k}w_{k|k-1}^{\left(j\right)}q_{k}^{\left(j\right)}\left(z\right)\frac{\langle \Psi_{k}^{1}\left[w_{k|k-1},Z_{k}\backslash \left\{z\right\}\right],p_{k|k-1}\rangle }{\langle \Psi_{k}^{0}\left[w_{k|k-1},Z_{k}\right],p_{k|k-1}\rangle }\frac{\langle 1,\kappa_{k}\rangle }{\kappa_{k}\left(z\right)}$$

(20) $$m_{k}^{\left(j\right)}\left(z\right)=m_{k|k-1}^{\left(j\right)}+K_{k}^{\left(j\right)}\left(z-\eta_{k|k-1}^{\left(j\right)}\right)$$

(21) $$P_{k}^{\left(j\right)}=\left[I-K_{k}^{\left(j\right)}H_{k}\right]P_{k|k-1}^{\left(j\right)}$$

(22) $$K_{k}^{\left(j\right)}=P_{k|k-1}^{\left(j\right)}H_{k}^{T}{\left[S_{k|k-1}^{\left(j\right)}\right]}^{-1}$$

Where |Z| denotes the element number of the set ***Z***, $p_{K,k}$ denotes the cardinality distribution of false alarm at time step *k*, $e_{j}\left(\cdot \right)$ denotes the elementary symmetric function of order *j*, $e_{j}(Z)=\sum_{S\subseteq Z,|S|=j}\left(\prod_{\zeta \in S}\zeta \right)$, $e_{0}(Z)=1$, $A_{l}^{j}=l!/(l-j)!$, and $Z_{k}\backslash \{z\}$ denotes the set $Z_{k}$ without element ***z***.

*Step* 3. *Managing Mixture Components*

Components with insignificant weights are removed, those with identical distributions are combined, and the total number of components is capped. Subsequently, the updated PHD at time step *k* is given as follows:

(23) $$v_{k}\left(x\right)=\sum_{i=1}^{J_{k}}w_{k}^{\left(i\right)}N\left(x;m_{k}^{\left(i\right)},P_{k}^{\left(i\right)}\right)$$

Where $w_{k}^{\left(i\right)}$ denotes the weight, and $m_{k}^{\left(i\right)}$ and $P_{k}^{\left(i\right)}$ denote the estimate of the state of the target and the MSEM at time step *k*, respectively.

*Step* 4. *State Estimation*

The *n* corresponding to the peak of the cardinality distribution $p_{k}\left(n\right)$ represents the estimated number of targets, denoted as ${\hat{N}}_{k}$. The $m_{k}^{\left(i\right)}$ associated with the components with the highest ${\hat{N}}_{k}$ weight in $v_{k}\left(x\right)$ corresponds to the estimated target states. The first element of the state estimate vector indicates the estimated bearing angle of the target. By inputting $p_{k}\left(n\right)$ and $v_{k}\left(x\right)$ into the subsequent time step, the GM-CPHD filter can be used to recursively estimate the states of targets to perform DOA tracking.

## 4. Saga–Husa CPHD Filter (SH-CPHD Filter) for Multi-Target DOA Tracking

The traditional DOA tracking methods typically assume that the measurement noise is stationary, implying a stable covariance matrix. However, due to the influence of the marine environment, measurement noise in real underwater scenes is often uncertain, meaning the covariance matrix varies with time. Consequently, the assumption of stationary measurement noise in traditional DOA tracking technology does not align with actual conditions, leading to decreased tracking performance. To enhance the robustness of multi-target DOA tracking methods, an improved Saga–Husa method is employed to estimate the covariance matrix of measurement noise in real time during tracking.

The classic Sage–Husa online noise estimator was firstly designed for a linear system with stable noise. In this classic estimator [17], it is assumed that noise is stable. Thus, data contributions at each time step are set equally in the estimation result. However, unstable noise due to uncertain environmental disturbances requires a different approach. Greater emphasis should be placed on the most recent information rather than historical data. Therefore, a weight coefficient $d_{k}$ is introduced. $d_{k}$ adjusts the ratio of the latest information’s contribution relative to historical information. As shown in Equation (26), the measurement noise is a scalar, not a vector. Consequently, the covariance matrix of the measurement noise reduces to the measurement noise variance. The modified Sage–Husa online noise estimator for uncertain measurement noise is given below.

(24) $${\hat{\sigma }}_{k}=\left(1-d_{k}\right){\hat{\sigma }}_{k-1}+d_{k}\left({\tilde{Z}}_{k}{\tilde{Z}}_{k}^{T}-H_{k}P_{k|k-1}H_{k}^{T}\right)$$

Where $H_{k}$ denotes the measurement matrix at tracking step *k*, given by Equation (14), and $P_{k|k-1}$ is the one-step prediction of the estimation error covariance matrix given by Equation (12). ${\tilde{Z}}_{k}$ is the innovation represented as follows:

(25) $${\tilde{Z}}_{k}=Z_{k}-H_{k}{\hat{X}}_{k|k-1}$$

The weight coefficient $d_{k}$ is expressed as follows:

(26) $$d_{k}=\left(1-b\right)/\left(1-b^{k}\right)$$

Where *b* is the forgetting factor and 0<b<1. Equation (26) illustrates that if *b* is closer to 1, the noise estimator, as defined in Equation (23), will prioritize information from the entire tracking period. Conversely, if *b* is closer to 0, the noise estimator will emphasize the most recent information. Therefore, adjusting the value of *b* dynamically changes the weight coefficient $d_{k}$, which impacts the performance of the modified Sage–Husa online noise estimator. If the measurement noise is seriously unstable due to the underwater environment, *b* should be set closer to 1 to maintain the stability in the tracking process.

The estimated variance of the measurement noise, derived using the modified Saga–Husa algorithm, is integrated into the CPHD filtering multi-target DOA tracking method, as detailed in Section 3. This results in the proposed Saga–Husa CPHD filter for the robust multi-target DOA tracking method (SH-CPHD) [18]. In the GM-CPHD filtering framework, a Gaussian mixture model represents multiple target states, with each Gaussian component corresponding to a target state. At each time step, multi-target measurements are obtained, necessitating the estimation of measurement noise variance for multiple targets using multiple measurements. As described in Section 3, the CPHD filter considers all possible combinations of measurements and components when updating the Gaussian components and their weights. Let the number of measurements be $m_{k}$ and the number of components be $J_{k|k-1}$. Then, the total number of updated components is $m_{k}\times J_{k|k-1}$. An updated component with a larger weight indicates higher confidence in that component. However, mismatched measurements used for updating components cause inaccuracies, leading to decreased weights for those components. The weight of the inaccurate component gradually decreases over time until it can no longer be trusted. During the Gaussian component update process, the Saga–Husa algorithm is employed to obtain $m_{k}\times J_{k|k-1}$ estimated variances of measurement noise for each updated component. Similarly, using the measurement noise variance from non-corresponding measurements to estimate the corresponding component weights causes those weights to gradually decrease until they are no longer reliable.

## 5. Smoothing SH-CPHD Filter for Robust Multi-Target DOA Tracking

### 5.1. Smoothing CPHD Filter

The CPHD backward smoothing recursive formula is derived as follows:

(27) $$\begin{array}{l} v_{t|k}(x)=v_{t|t}[\left(1-p_{S,k}\right)\frac{\gamma_{k}^{1}v_{t+1|k},Z_{k}],\rho_{t+1}|k}{\gamma_{k}^{0}\left[v_{t+1|k},Z_{k}\right],\rho_{t+1}|k}+\\ p_{S,k}\int \frac{\gamma_{k}^{1}\left[v_{t+1|k},Z_{k}\setminus \{z\}\right],\rho_{t+1|k}}{\gamma_{k}^{0}\left[v_{t+1}|k,Z_{k}\right],\rho_{t+1|k}}\frac{f_{k|k+1}(x|\zeta )v_{t+1|k}}{v_{t+1|t}}dx_{t+1}] \end{array}$$

where t=k−1,k−2,⋯,k−L(k>t), and *L* denotes the lag time of smoothing algorithms. The intensity function of the CPHD filter at step *t* is represented by $v_{t+1|t}$. The intensity functions of the CPHD filter at steps *t* and t+1 are denoted by $v_{t|k}$ and $v_{t+1|k}$. The predicted intensity function is indicated by $v_{t+1|t}$.

According to Equation (27), the updated intensity function of the CPHD filter at step *t* and step t+1 are given as follows:

(28) $$v_{t|t}(x)=\sum_{i=1}^{J_{t}|t}\omega_{t|t}^{i}N\left(x_{t};m_{t|t}^{i},P_{t|t}^{i}\right)$$

(29) $$v_{t+1|k}(x)=\sum_{j=1}^{{J_{t+1}|}^{k}}\omega_{t+1|k}^{j}N\left(x_{t+1};m_{t+1|k}^{j},P_{t+1|k}^{j}\right)$$

Substituting Equations (28) and (29) into Equation (27), the smoothed PHD is obtained and given as follows:

(30) $$\begin{array}{l} v_{t|k}(x)=\left(1-p_{S,k}\right)\frac{\Psi_{k}^{1}\left[\omega_{t+1|k},Z_{k}\right],\rho_{t+1|k}}{\Psi_{k}^{o}\left[\omega_{t+1|k},Z_{k}\right],\rho_{t+1|k}}\times \sum_{i=1}^{J_{t|t}}\omega_{t|t}^{i}N\left(x_{t};m_{t|t}^{i},P_{t|t}^{i}\right)+\\ \sum_{i=1}^{J_{t|1}}\sum_{j=1}^{J_{t+1}|k}\omega_{t|t}^{i}\omega_{t+1|k}^{j}N\left(x_{t};m_{t|t}^{i},P_{t|t}^{i}\right)\times p_{S,k}\int \frac{\Psi_{k}^{1}\left[\omega_{t+1|k},Z_{k}\right],\rho_{t+1|k}}{\Psi_{k}^{0}\left[\omega_{t+1|k},Z_{k}\right],\rho_{t+1|k}}\frac{N\left(x_{t+1};m_{t+1|k}^{j},P_{t+1|k}^{j}\right)}{v_{t+1|t}}\\ \times N\left(x_{t+1};F_{t}x_{t},F_{t}P_{t|t}^{i}F_{t}^{T}+Q_{t}\right)dx_{t+1} \end{array}$$

The GM implementation form of the above smoothed filter is as follows:

(31) $$v_{t|k}(x)=\left(1-p_{S,k}\right)\sum_{i=1}^{t_{t1}}\omega_{t|t}^{i}N\left(x_{t};m_{t|t}^{i},P_{t|t}^{i}\right)+p_{S,k}\sum_{i=1}^{J_{t1}}{\sum_{j=1}^{J_{t+1}}}^{k}{\omega_{t}^{i,j}}_{k}N\left(x_{t+1};{m_{t}^{i,j}}_{k},P_{t{\}}_{k}^{i,j}}\right)$$

(32) $$\Psi_{k}^{u}[\omega ,Z](n)=\sum_{j=0}^{\min(1z|,m)}\left(\left(|Z|-j)!p_{K,k}(|Z|-j\right)\right)\times P_{j+u}^{n}\frac{1-p_{D,k},\omega^{n-(j+\omega )}}{{\langle 1,\omega \rangle }^{j+u}}e_{j}\left(\Lambda_{k}(\omega ,Z)\right)$$

Where

(33) $$\Lambda_{k}(\omega ,Z)=\left\{\frac{1,\kappa_{k}}{\kappa_{k}(z)}p_{D,k}\omega^{T}q_{k}(z):z\in Z\right\}$$

(34) $$m_{t|k}^{i,j}=m_{t|t}^{i}+A_{t}^{i,j}\left(m_{t+1|k}^{j}-F_{t}m_{t,j}^{i}\right)$$

(35) $$P_{t|k}^{i,j}=P_{t|t}^{i}+A_{t}^{i,j}\left(P_{t+1|k}^{j}-\left(F_{t}P_{t||}^{i}F_{t}^{T}+Q_{t}\right)\right){\left(A_{t}^{i,j}\right)}^{T}$$

(36) $$A_{t}^{i,j}=P_{t|}^{i}F_{t}{\left(F_{t}P_{t|,}^{i}F_{t}^{T}+Q_{t}\right)}^{-1}$$

And the weight of the Gaussian mixture component is as follows:

(37) $$\omega_{t}^{i,j}=\frac{\omega_{t+1|k}^{j}\omega_{t}^{i}|t,\left(m_{t+1|k}^{j};F_{t}m_{t|t}^{i},F_{t}P_{t|1}^{i}F_{t}^{T}+Q_{t}\right)}{\gamma_{t+1}\left(m_{t+1|k}^{j}\right)+\sum_{l=1}^{J_{t=1}}\omega_{t|t}^{l}N\left(m_{t+1}^{j};F_{t}P_{t|l}^{l}F_{t}^{T}+Q_{t}\right)}$$

The equation describes the smoothing operation for the GM-CPHD filter. Similarly to forward CPHD filtering, backward smoothing also experiences an increase in the number of components. This issue can be addressed by applying any Gaussian mixture reduction method.

### 5.2. Algorithm of Smoothing SH-CPHD Filter

As mentioned in Section 4, the Saga–Husa algorithm is proposed to estimate the variance of the measurement noise within the CPHD filter framework to enhance tracking robustness under uncertain measurement noise conditions. By incorporating the smoother described in the equations into the SH-CPHD filter, the smoothed SH-CPHD filter is proposed. This approach improves the robustness of underwater DOA tracking when faced with uncertain measurement noise. The algorithm for the smoothed SH-CPHD filter is presented as Algorithm 1.
**Algorithm 1:** Smoothing SH-CPHD filter for robust multi-target DOA tracking1. Initialize the components of Gaussian mixture model ${\left\{w_{0}^{\left(i\right)},m_{0}^{\left(i\right)},P_{0}^{\left(i\right)}\right\}}_{i=1}^{J_{0}}$ and cardinalized distribution $p_{0}\left(n\right)$; For k=1:K Prediction: 2. Calculate the predicted cardinalized distribution $p_{k|k-1}\left(n\right)$ according to Equation (3); 3. Calculate the components of survival targets: For $i=1:J_{k-1}$ $w_{s,k|k-1}^{\left(i\right)}=p_{s,k}w_{k-1}^{\left(i\right)}$, $m_{s,k|k-1}^{\left(i\right)}=F_{k-1}m_{k-1}^{\left(i\right)}$, $P_{s,k|k-1}^{\left(i\right)}=G_{k}\sigma_{q}^{2}G_{k}^{T}+F_{k-1}P_{k-1}^{\left(i\right)}F_{k-1}^{T}$; End 4. Add the components of birth targets ${\left\{w_{\gamma ,k}^{\left(i\right)},m_{\gamma ,k}^{\left(i\right)},P_{\gamma ,k}^{\left(i\right)}\right\}}_{i=J_{k-1}+1}^{i=J_{k-1}+J_{\gamma ,k}}$; 5. Express the predicted GM components as ${\left\{w_{k|k-1}^{\left(i\right)},m_{k|k-1}^{\left(i\right)},P_{k|k-1}^{\left(i\right)}\right\}}_{i=1}^{i=J_{k|k-1}}$, where $J_{k|k-1}=J_{k-1}+J_{\gamma ,k}$; Update: 6. Update the cardinality distribution to $p_{k}\left(n\right)$ by using Equation (10); 7. The DOA estimation method is used to process these extracted signals to estimate the bearing angle of the target, and the bearing angle measurement set $Z_{k}^{m}=\left\{z_{1},z_{2},\cdots z_{m_{k}}\right\}$ is obtained. 8. Update GM components of targets: For $i=1:J_{k|k-1}$ $w_{k}^{\left(i\right)}=\frac{\langle \Psi_{k}^{1}\left[w_{k|k-1},Z_{k}\right],p_{k|k-1}\rangle }{\langle \Psi_{k}^{0}\left[w_{k|k-1},Z_{k}\right],p_{k|k-1}\rangle }\left(1-p_{D,k}\right)w_{k-1}^{\left(i\right)}$, $m_{k}^{\left(i\right)}=m_{k|k-1}^{\left(i\right)}$, $P_{k}^{\left(i\right)}=P_{k|k-1}^{\left(i\right)}$; end For $m=1:m_{k}$  For $i=1:J_{k|k-1}$  $S_{k|k-1}^{\left(i\right)}=H_{k}P_{k|k-1}^{\left(i\right)}H_{k}^{T}+{\hat{\sigma }}_{r,k}^{2}$, $K_{k}^{\left(i\right)}=P_{k|k-1}^{\left(i\right)}H_{k}^{T}{\left[S_{k|k-1}^{\left(i\right)}\right]}^{-1}$,  $w_{k}^{\left(J_{k|k-1}+(m-1)m_{k}+i\right)}=p_{D,k}w_{k|k-1}^{\left(i\right)}q_{k}^{\left(i\right)}\left(z_{m}\right)\frac{\langle \Psi_{k}^{1}\left[w_{k|k-1},Z_{k}\backslash \left\{z_{m}\right\}\right],p_{k|k-1}\rangle }{\langle \Psi_{k}^{0}\left[w_{k|k-1},Z_{k}\right],p_{k|k-1}\rangle }\frac{\langle 1,\kappa_{k}\rangle }{\kappa_{k}\left(z_{m}\right)}$,  $m_{k}^{\left(J_{k|k-1}+(m-1)m_{k}+i\right)}=m_{k|k-1}^{\left(i\right)}+K_{k}^{\left(i\right)}\left(z_{m}-\eta_{k|k-1}^{\left(i\right)}\right)$,  $P_{k}^{\left(J_{k|k-1}+(m-1)m_{k}+i\right)}=\left[I-K_{k}^{\left(i\right)}H_{k}\right]P_{k|k-1}^{\left(i\right)}$;  End End 9. $J_{k|k}=J_{k|k-1}+J_{k|k-1}m_{k}$, prune, merge and limit the $J_{k|k}$ components to obtain new $J_{k}$ components; 10. Express the updated GM components as ${\left\{w_{k}^{\left(i\right)},m_{k}^{\left(i\right)},P_{k}^{\left(i\right)}\right\}}_{i=1}^{i=J_{k}}$; 11. The estimate of target number ${\hat{N}}_{k}$ is the *n* corresponding to the maximum of $p_{k}\left(n\right)$; 12. The $m_{k}^{\left(i\right)}$ corresponding to the components with the largest ${\hat{N}}_{k}$ weights are the estimates of target states. End Smoothing: 13. Initialize T=K, ${m_{T|k}|}_{j=1}^{j=J_{T}}={m_{T}|}_{j=1}^{j=J_{T}}$, ${\omega_{T|k}|}_{j=1}^{j=J_{T}}={\omega_{T}|}_{j=1}^{j=J_{T}}$, ${P_{T|k}|}_{j=1}^{j=J_{T}}={P_{T}|}_{j=1}^{j=J_{T}}$; For t=T−1:1 14. Smooth the PHD For $j=1:J_{t+1}$ For $i=1:J_{t}$ $m_{t|k}^{i,j}=m_{t|t}^{i}+A_{t}^{i,j}\left(m_{t+1|k}^{j}-F_{t}m_{t,j}^{i}\right)$ $P_{t|k}^{i,j}=P_{t|t}^{i}+A_{t}^{i,j}\left(P_{t+1|k}^{j}-\left(F_{t}P_{t||}^{i}F_{t}^{T}+Q_{t}\right)\right){\left(A_{t}^{i,j}\right)}^{T}$ $A_{t}^{i,j}=P_{t|}^{i}F_{t}{\left(F_{t}P_{t|,}^{i}F_{t}^{T}+Q_{t}\right)}^{-1}$ $\omega_{t}^{i,j}=\frac{\omega_{t+1|k}^{j}\omega_{t}^{i}|t,\left(m_{t+1|k}^{j};F_{t}m_{t|t}^{i},F_{t}P_{t|1}^{i}F_{t}^{T}+Q_{t}\right)}{\gamma_{t+1}\left(m_{t+1|k}^{j}\right)+\sum_{l=1}^{J_{t=1}}\omega_{t|t}^{l}N\left(m_{t+1}^{j};F_{t}P_{t|l}^{l}F_{t}^{T}+Q_{t}\right)}$ 15. $J_{k|k}=J_{k|k-1}+J_{k|k-1}m_{k}$, prune, merge and limit the $J_{k|k}$ components to obtain new $J_{k}$ components; End End

## 6. Simulations

### 6.1. Simulation Scenario

**Figure 1:**
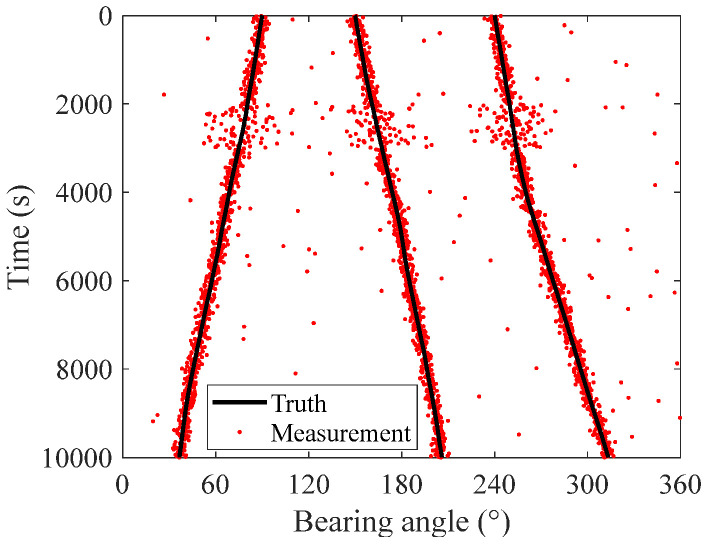
Simulated trajectories and measurements of the targets.

Simulations are conducted to verify the performance of the proposed smoothing SH-CPHD filter for the robust underwater multi-target DOA tracking method. It is assumed that a hydrophone array is positioned underwater. Within the detection area of the hydrophone array, three underwater targets are continuously emitting acoustic signals. The target directions’ trajectories are simulated using the kinematic model described by Equation (1). The total tracking duration is 1000 s, with a 1 s interval between each time step. The initial states of the targets are ${\left[-90,-0.02\right]}^{T}$, ${\left[150,0.01\right]}^{T}$, and ${\left[240,0.015\right]}^{T}$. The process noise covariance matrix is $2\times 10^{-4}{}^{\circ}/s^{2}$. The black solid lines in Figure 1 illustrate the simulated trajectories of the target directions, and the red dots represent measurements.

The standard derivation of the measurement noise is set to 5°. Considering the uncertain measurement noise caused by unknown ocean environments in real underwater tracking scenarios, standard deviation is increased tenfold, from 2000 s to 3000 s, to simulate the impact of underwater environmental noise on the measurements. Based on this configuration, the measurement data are simulated and used to evaluate the performance of the proposed smoothing SH-CPHD filter for robust underwater multi-target DOA tracking. For comparison, tracking results using the Minimum Variance Distortionless Response (MVDR) method [19], PHD filter [11], CPHD filter [20], and SH-CPHD filter [18] are also provided.

To statistically assess the tracking performance of the proposed method, 500 Monte Carlo simulations are conducted. An error metric is needed to measure the difference between the real target set and the estimated target set. However, due to the varying number of elements in the real and estimated target sets, the root mean square metrics (such as root-mean-square deviation) are considered unsuitable. Instead, the optimal sub-pattern assignment (OSPA) error is used to evaluate the performance of multi-target DOA tracking methods. The OSPA error is defined as follows [21]:

(38) $$\{\begin{array}{ll} d_{p}^{\left(c\right)}\left(X,Y\right)={\left(\frac{1}{n}\underset{\pi \in \Pi }{\min}\sum_{i=1}^{m}d^{\left(c\right)}{\left(x_{i},y_{\pi \left(i\right)}\right)}^{p}+c^{p}\left(n-m\right)\right)}^{1/p}, & m\leq n\\ d_{p}^{\left(c\right)}\left(X,Y\right)=d_{p}^{\left(c\right)}\left(Y,X\right), & m>n \end{array}$$

The OSPA error, denoted as $d_{p}^{\left(c\right)}\left(X,Y\right)$, measures the difference between two random finite sets RFS $X=\left\{x_{1},x_{2},\ldots ,x_{m}\right\}$ and $Y=\left\{y_{1},y_{2},\ldots ,y_{n}\right\}$. Here, Π represents the set consisting of *m* elements taken from {1,2,…,n}. The Euclidean distance d(x,y) between *x* and *y* is denoted as $d^{\left(c\right)}\left(x,y\right)=\min\left\{d\left(x,y\right),c\right\}$. The truncation parameter *c* is set to 5, and the OSPA metric order parameter *p* is set to 1. A smaller OSPA error denotes better tracking accuracy.

### 6.2. Verification of the Smoothing SH-CPHD Filter for Robust Multi-Target DOA Tracking

**Figure 2:**
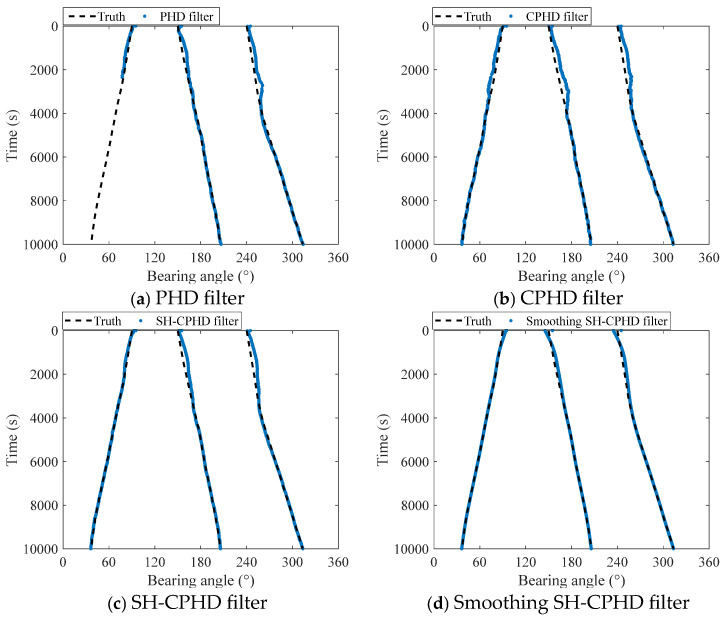
DOA tracking results of the PHD filter, CPHD filter, SH-CPHD filter, and smoothing SH-CPHD filter.

The DOA tracking results obtained using the MVDR, PHD filter, CPHD filter, SH-CPHD filter, and the proposed smoothing SH-CPHD filter for the robust multi-target DOA tracking method are shown in Figure 2. As illustrated, all methods provide stable tracking for all three targets when the measurement noise variance is constant from 0 to 2000 s. However, when the measurement noise variance increases due to the unknown underwater environment noise from 2000 to 3000 s, the MVDR is almost invalid. The PHD filter and CPHD filter produce fluctuating tracking trajectories and may even lose track of the targets, leading to higher tracking errors. The SH-CPHD filter achieves higher tracking accuracy than the PHD and CPHD filters by estimating the measurement noise variance in real time. The proposed smoothing SH-CPHD filter for the robust multi-target DOA tracking method provides the stable tracking of all three targets throughout the whole tracking process. The tracking trajectory matches the real trajectory well, even during the period of increased measurement noise variance. The reason is that the backward smoothing technique utilized by the proposed method eliminates the error generated during forward filtering. Consequently, the precision of tracking is developed, even in the scenario of increasing measurement noise covariance matrix results due to unknown underwater environmental noise.

**Figure 3:**
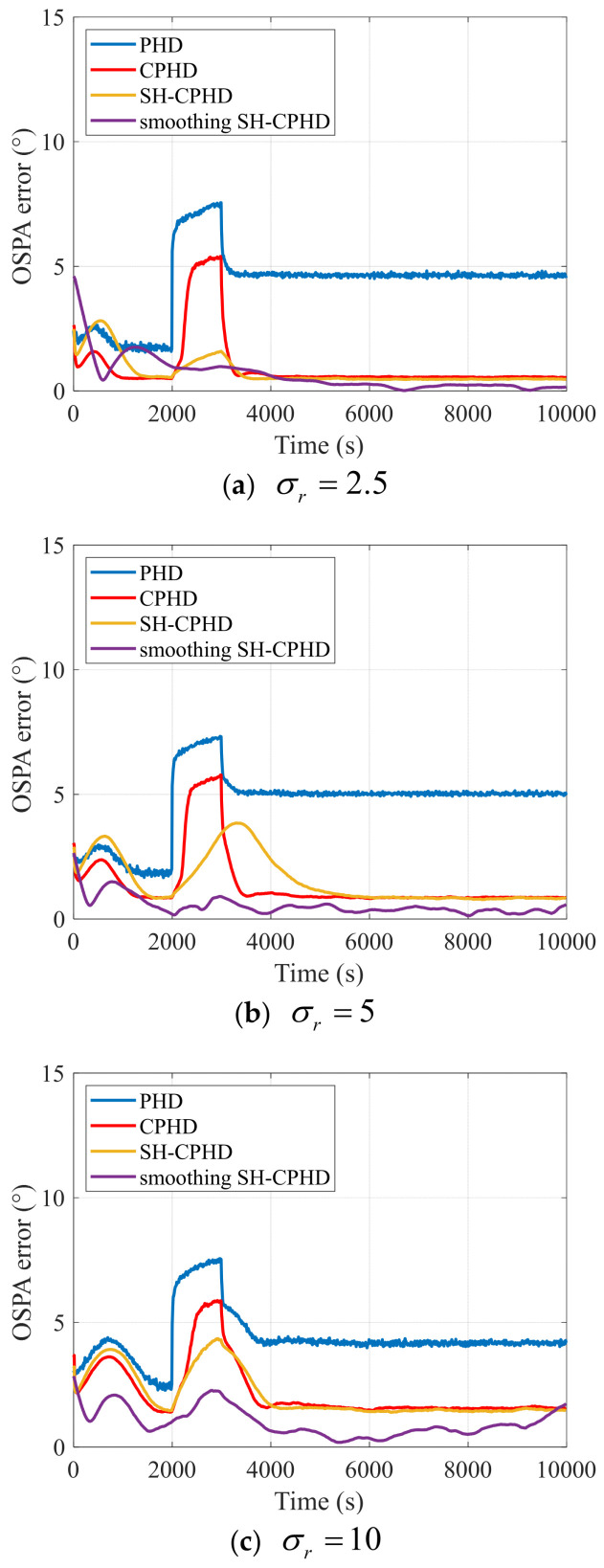
Average OSPA error of the Monte Carlo simulation results of the MVDR, PHD filter, CPHD filter, and smoothing SH-CPHD filter in the scenario where $\sigma_{r}=2.5,5,10$.

To further evaluate the tracking performance of the proposed method statistically, the Monte Carlo simulation results are presented. To compare the tracking performance under measurement noise with different covariance matrices, the standard deviation of the measurement noise is set to 2.5°, 5°, and 10°. The average OSPA error of the Monte Carlo simulation results at each time step is shown in Figure 3. The average OSPA error during the period of increasing measurement noise covariance is shown in Table 1.

Figure 3 shows that the proposed smoothing SH-CPHD filter for the robust multi-target DOA tracking method provides high-precision tracking when $\sigma_{r}$ is 2.5°, 5°, and 10°, respectively. As $\sigma_{r}$ increases, the OSPA errors of the MVDR, PHD filter, CPHD filter, SH-CPHD filter, and the proposed smoothing SH-CPHD filter for the robust multi-target DOA tracking method all increase. From 0 to 600 s and 800 s to 1000 s, when the measurement noise covariance matrix is stable, the estimation errors of all methods are small. The MVDR method provides the largest DOA tracking error. Due to the utilization of the kinematic model, the PHD filter, CPHD filter, SH-CPHD filter, and smoothing SH-CPHD filter show improved performance compared to the MVDR. Because the cardinalized distribution is introduced in the CPHD filter in the base of PHD filter, the tracking performance of the CPHD-filter-based methods is improved compared to the PHD filter. During the period from 600 s to 800 s, when the measurement noise covariance matrix increases, the OSPA errors of the MVDR, PHD filter, and CPHD filter significantly increase compared to the period when the measurement noise covariance is stable. Meanwhile, the OSPA errors of the SH-CPHD filter are smaller than those of the PHD filter and CPHD filter, and the proposed smoothing SH-CPHD filter provides more accurate tracking than the SH-CPHD filter due to the backward smoothing technique. The results presented in Table 1 further demonstrate the robustness of the proposed method in scenarios of uncertain measurement noise caused by unknown marine environmental conditions.

Furthermore, to further validate the robustness of the algorithm, we performed simulations under various initial error conditions and present the corresponding OSPA plots. In this scenario, a set of different b values was established, and multiple Monte Carlo simulation experiments were conducted for each parameter combination. By comparing the estimation errors, convergence speeds, and robustness indices across the different parameters, we reveal the sensitivity and impact of b on the estimator’s performance.

**Figure 4:**
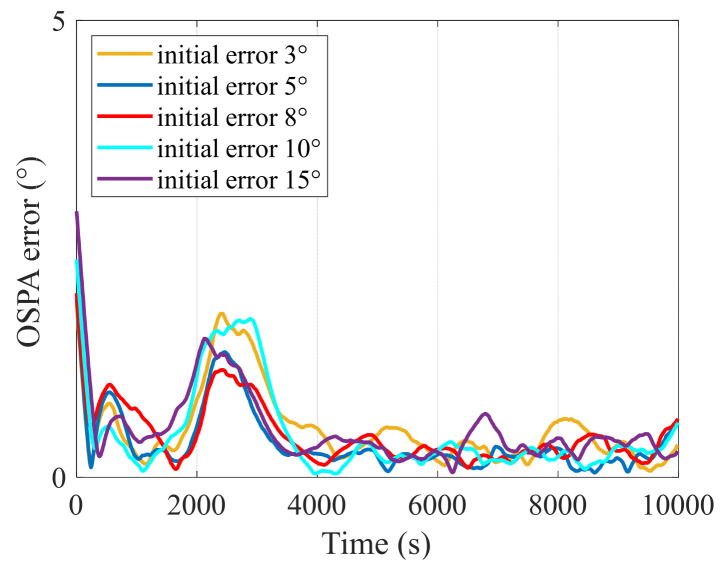
OSPA errors under different initial error conditions.

Figure 4 illustrates the OSPA errors under different initial error conditions. As observed, variations in the initial error produce only minor changes in the initial OSPA error, and as time progresses, the OSPA results for different initial errors converge closely. This indicates that the algorithm exhibits strong robustness to initial error. This robustness is attributed to the CPHD filter’s dual estimation approach, which simultaneously propagates the probability density function of the target states and the probability distribution of the target count. Consequently, even if there is a significant deviation in the initial estimates of the target count or state, the CPHD filter can swiftly rectify these errors. Moreover, the SH algorithm within the proposed method reduces the influence of the initial error during the forward filtering process, while the smoothing algorithm further mitigates its effect during backward smoothing.

**Figure 5:**
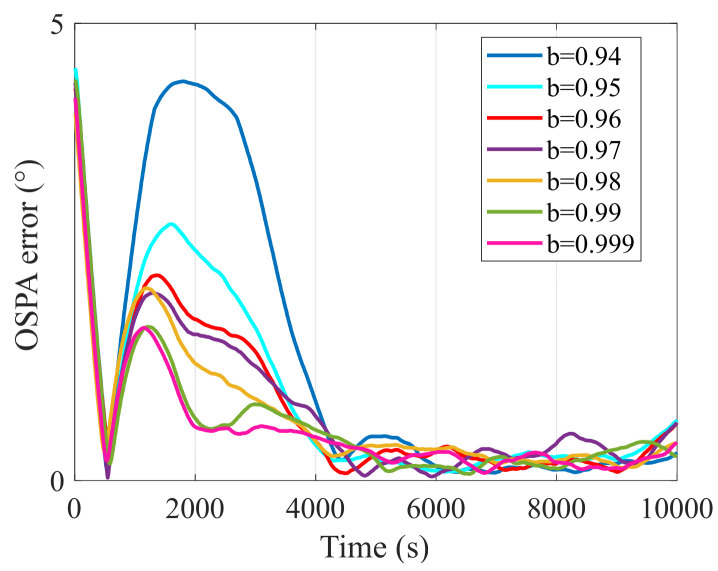
OSPA errors under different forgetting factors.

Figure 5 shows the OSPA results obtained under different forgetting factors b. As observed, with an increase in the forgetting factor, the performance in the presence of outliers gradually improves, which is further corroborated by the data in Table 2. This improvement can be attributed to the fact that a larger forgetting factor implies that the influence of new data is relatively reduced during each update, thereby reinforcing the memory of previously collected observations. The smoothing effect of historical data diminishes the estimation fluctuations caused by one or a few new data points, leading to a more stable noise covariance estimation and making the filter less susceptible to disturbances from abnormal measurements or sudden noise spikes. However, when the historical data are also highly unstable, an excessively large forgetting factor may result in suboptimal performance, even under relatively stable observations.

In the CPHD algorithm, both the detection probability and false alarm rate have a significant impact on tracking performance. In underwater complex environments, these two parameters are often uncertain; therefore, we conducted simulation experiments comparing different detection probabilities and false alarm rates, and we presented the results using the OSPA metric to explore their influence on the algorithm.

**Figure 6:**
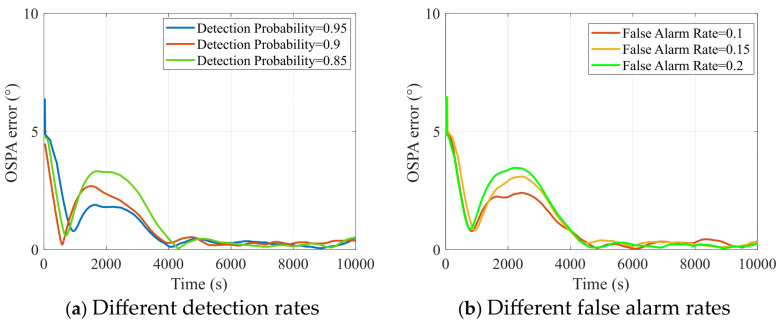
OSPA errors under different detection rates and false alarm rates.

Figure 6 demonstrates the OSPA errors under different false alarm and detection rates. As observed in Figure 6a, in the non-stationary measurement regime, the OSPA error increases significantly with an increase in detection probability. This phenomenon occurs because, when the measurement noise is substantially elevated, maintaining a high detection probability ensures that the target is not missed. The continuous input of observations allows the filter to repeatedly update and correct the model, thereby reducing the tracking bias or loss risk that may arise from prediction errors.

In Figure 6b, it can be seen that as the false alarm rate increases, the OSPA error also rises continuously. This is because, in the proposed algorithm, the false alarm rate is defined as the clutter rate, that is, the probability that a given measurement is not generated by any target. Evidently, as the clutter rate increases, the algorithm’s accuracy decreases. However, this degradation is confined to periods with non-stationary measurements; during other times, the proposed algorithm maintains strong robustness to variations in the clutter rate. This occurs because a higher clutter rate further exacerbates the non-stationary measurement phenomenon.

Based on the preceding simulations, the proposed algorithm has been thoroughly analyzed and validated. We now proceed to evaluate its real-world performance using measured data.

## 7. Experimental Results

### 7.1. Experimental Setup and Description

**Figure 7:**
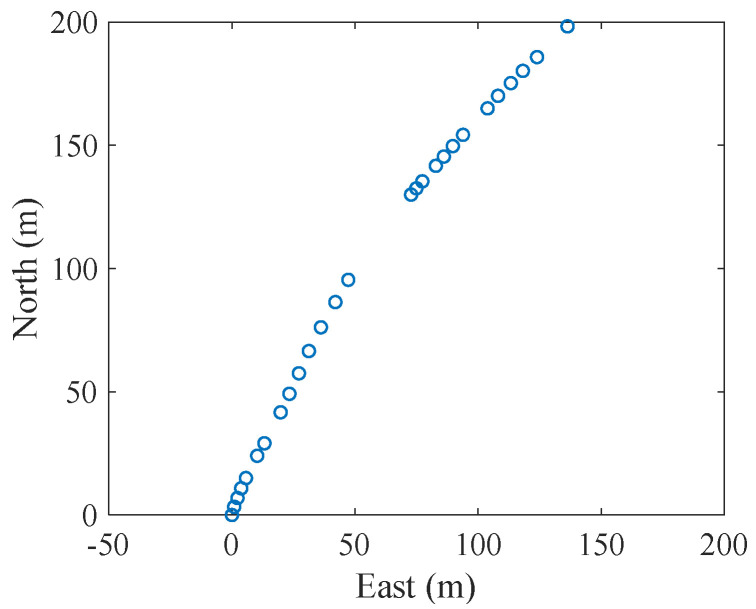
Position of the element.

The open experimental dataset SWellEx96 [22] was used to verify the tracking performance of the proposed smoothing SH-CPHD filter for robust DOA tracking in a real underwater tracking scenario. The experiment was performed by the United States Marine Physical Laboratory from 10 to 18 May 1996, approximately 12 km from the tip of Point Loma near San Diego, California. The data from the north horizontal linear array of the S59 event of the experiment were used in this section. Experimental data corresponding to 900 s were used to test the proposed method in this section. Before the experiment, a CTD was used to obtain the sound velocity in the experimental area. The experimental ship towed a continuous sound source at a depth of 54 m, at a speed of five knots, and sailed north, which is referred to as target 1 in this paper. In addition, an uncooperative ship sailed from northwest to southeast with continuous radiating noise, which is referred to as target 2 in this paper. The radar system of the experimental ship recorded the distance and bearing of the uncooperative ship and derived its latitude and longitude. The horizontal linear array was placed on the seafloor at a depth of 213 m and continuously recorded the received acoustic signal at a sampling frequency of 3276.8 Hz. The horizontal array consisted of 32 elements, 27 of which provided effective data. The positions of the effective elements are shown in Figure 7, where the position of the first element was taken as the origin of the coordinates. Figure 7 shows that the elements are not arranged in a straight line but instead follow a slight curve. Therefore, the port and starboard ambiguity problem of the linear array was partly avoided.

### 7.2. Verification of the Smoothing SH-CPHD Filter for Robust Multi-Target DOA Tracjing by Using Experimental Data

**Figure 8:**
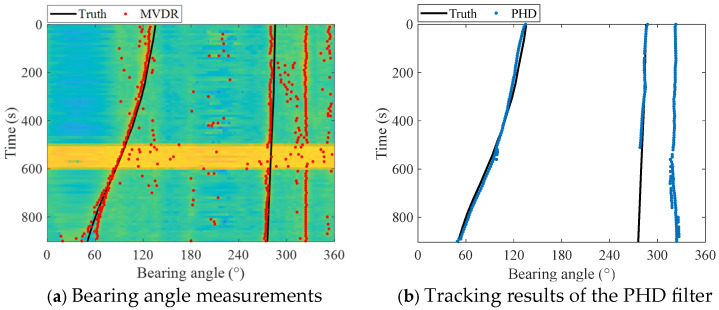

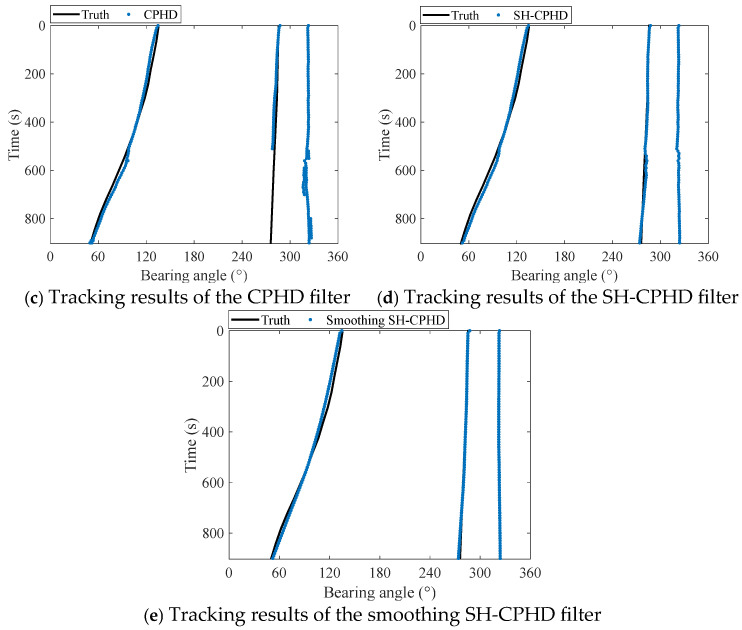
Tracking results of the MVDR, PHD filter, CPHD filter, SH-CPHD filter, and smoothing SH-CPHD filter.

According to the CTD data, the sound velocity was set to 1493 m/s. The true bearing angles of the two targets are indicated by the black line in Figure 8a. The bearing time recording (BTR) obtained by using the MVDR is shown in the background of Figure 8a. The bearing angles corresponding to the peaks of the spectrum were extracted at each moment, and the obtained measurements of the bearing angles of the targets are represented by red dots in Figure 8a. Since the power of measurement noise hardly varies in the experiment, the superior performance of the proposed smoothing SH-CPHD filter for robust multi-target DOA tracking cannot be extensively shown. Thus, to test the robustness of the proposed underwater multi-target DOA tracking method in scenarios of uncertain measurement noise, a period of high-power noise was added to the experimental data, from 500 s to 600 s. Figure 8a shows the trajectories of three targets. The bearing angle of target 1 moves from 135° to 50°, the bearing angle of target 2 moves from 285° to 275°, and the bearing angle of an unknown uncooperative target remains near 320°, which is referred to as target 3 in this paper.

The bearing angle measurements were processed using the proposed smoothing SH-CPHD filter for robust multi-target DOA tracking, and the results of the PHD filter, CPHD filter, and SH-CPHD filter are also provided for comparison. The process noise variance of the CV model was set to $2.5\times 10^{-4}$. The detection probability and false alarm probability were set to 0.9 and 0.1, respectively. The bearing angle measurement noise variance was set to 25.

**Figure 9:**
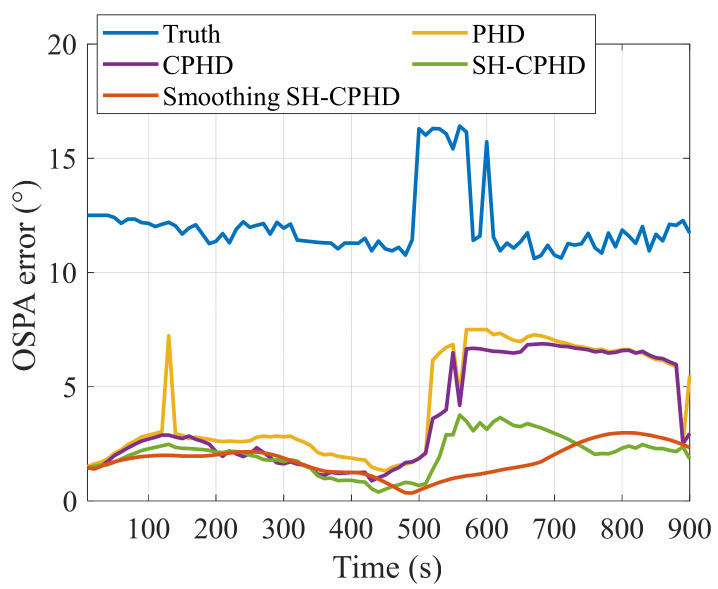
OSPA errors of the MVDR, PHD filter, CPHD filter, and smoothing SH-CPHD filter.

The tracking results of PHD filter, CPHD filter, SH-CPHD filter, and smoothing SH-CPHD filter are shown in Figure 8b–d, and the OSPA errors are shown in Figure 9. The average OSPA error tracking steps are shown in Table 2. Figure 8b–d shows that all multi-target DOA tracking methods carry out stable tracking of the three targets, and the tracking trajectories are consistent with the real trajectories. Figure 9 and Table 3 indicate that the PHD filter, CPHD filter, SH-CPHD filter, and smoothing SH-CPHD filter significantly reduce OSPA errors based on measurements, with the OSPA errors of the smoothing SH-CPHD filter being slightly smaller than those of the PHD filter, CPHD filter, and SH-CPHD filter.

Figure 7 and Figure 9 demonstrate that the PHD filter and CPHD filter conduct stable tracking of the bearing angles of three targets when the measurement noise variance is stationary from 0 to 500 s. However, when the measurement noise variance increases from 500 s to 600 s, the assumption of fixed measurement noise variance in the PHD filter and CPHD filter becomes inconsistent with the actual increasing measurement noise variance, resulting in an inaccurate Kalman filter gain. Consequently, the PHD filter and CPHD filter exhibit fluctuating tracking trajectories or even fail to track the targets, leading to an increase in OSPA error. The OSPA error of the SH-CPHD filter for robust multi-target DOA tracking is significantly less than that of the PHD filter and CPHD filter. The reason is that the SH-CPHD filter estimates the measurement noise variance in real time tracking, so the Kalman filter gain calculated by using the estimate of measurement noise variance is more accurate. The proposed smoothing SH-CPHD filter employs a backward smoothing technique to eliminate errors in the forward filter. Therefore, the increasing measurement noise variance hardly affects the performance of the SH-CPHD filter, and robust DOA tracking with uncertain measurement noise is achieved. Table 3 again proves the superiority of the proposed smoothing SH-CPHD filter in terms of robustness.

## 8. Conclusions

The problem of robust multi-target underwater DOA tracking in scenarios of uncertain measurement noise is studied in this paper. The Saga–Husa noise estimation technique and backward smoothing technique are employed during the CPHD-filter-based multi-target DOA tracking procedure, forming what we refer to as the smoothing SH-CPHD filter. In the presence of uncertain measurement noise, the state estimation process tends to become less reliable. To address this, the Saga–Husa noise estimation technique is used to estimate the variance of the measurement noise in real time, thereby enhancing the reliability of the filtering process. This noise variance estimate is dynamically incorporated into the tracking algorithm to adapt to variations in the measurement noise. Subsequently, the backward smoothing technique is applied to refine the forward filter results, further reducing tracking errors and improving overall estimation accuracy. By adaptively estimating the measurement noise and refining tracking outcomes through backward smoothing, the proposed smoothing SH-CPHD filter effectively enhances the robustness of underwater multi-target DOA tracking under uncertain noise conditions. Based on our simulation and experimental results, the proposed method is a viable approach for performing DOA tracking missions, particularly in challenging underwater environments characterized by uncertain measurement noise.

## Figures and Tables

**Table 1:** Average OSPA error of the MVDR, PHD filter, CPHD filter, and smoothing SH-CPHD filter when the measurement noise covariance matrix is increasing.

	MVDR	PHD	CPHD	SH-CPHD	Smoothing SH-CPHD Filter
Average OSPA error when $\sigma_{r}=2.5{}^{\circ}$ (°)	5.70	2.46	1.30	0.71	0.48
Average OSPA error when $\sigma_{r}=5{}^{\circ}$ (°)	6.05	3.84	1.58	0.72	0.56
Average OSPA error when $\sigma_{r}=10{}^{\circ}$ (°)	6.37	5.01	3.02	0.72	0.61

**Table 2:** Average OSPA errors under different forgetting factors.

b	0.94	0.95	0.96	0.97	0.98	0.99	0.999
Average OSPA Error (°)	1.33	0.95	0.77	0.73	0.69	0.59	0.54

**Table 3:** Average OSPA errors of the tracking results of the MVDR, PHD filter, CPHD filter, and smoothing SH-CPHD filter in one tracking step.

	MVDR	PHD	CPHD	SH-CPHD	Smoothing SH-CPHD
Average OSPA error (°)	4.26	4.22	3.71	2.49	2.06

## Data Availability

The data presented in this study are openly available in Swellex-96 at [Swellex-96] [https://swellex96.ucsd.edu/, accessed on 7 April 2025].
